# Professional perspectives on barriers to accessing maternity care in England: a qualitative study

**DOI:** 10.1186/s12884-026-08745-7

**Published:** 2026-02-10

**Authors:** Kerry Brennan-Tovey, Kausiki Sarma, Dafni Lima, Abimbola Ayorinde, Heather Brown, Oluwaseun B Esan, David Gardiner, Ruth Kipping, Nicola Heslehurst, Maria Raisa Jessica Aquino, Judith Rankin

**Affiliations:** 1 Population Health Sciences Institute, Faculty of Medical Sciences, Newcastle University, Baddiley-Clark Building, Richardson Road, Newcastle upon Tyne, NE2 4AX UK; 2 Warwick Medical School, University of Warwick, Gibbet Hill Road, Coventry, CV4 7AL UK; 3 Division of Health Research, Faculty of Health and Medicine, Health Innovation One, Lancaster University, Sir John Fisher Drive, Lancaster, LA1 4AT UK; 4 NIHR School for Public Health Research Launching Fellow, Department of Public Health, Policy and Systems, University of Liverpool, Waterhouse Building (2nd Floor, Block F), 1-5 Brownlow Street, Liverpool, L69 3GL UK; 5 Office for Health Improvement and Disparities, Stella House, Goldcrest Way, Newburn Riverside, Newcastle, NE15 8NY UK; 6 Population Health Sciences, Bristol Medical School, University of Bristol, Bristol, BS8 2PS UK; 7 Durham Law School, Durham University, Durham, DH1 3LE UK

**Keywords:** Poverty, Barriers, Access, Professionals, Maternity

## Abstract

**Background:**

Women living on low income in England are at an increased risk of experiencing stillbirth, neonatal death, preterm birth, low birth weight and maternal mortality. Women with poor access to financial, educational, and social and health resources engage less with health and care services throughout their pregnancy, due to social stressors, low health literacy, digital exclusion, lack of support, language barriers, transport difficulties, and stigma and judgement from healthcare professionals. Existing evidence documents the experiences of women facing socioeconomic disadvantage, little is known about how healthcare professionals understand and respond to these barriers. The aim of this qualitative study was to explore professionals’ perceptions of the barriers pregnant women living on low income face when accessing maternity care.

**Methods:**

Data were collected through one-to-one semi-structured interviews with professionals (i.e., midwives, health visitors, Voluntary, Community and Social Enterprise (VCSE) practitioner) working in the NHS, local authority or VCSE organisations in the North East of England. Purposive snowballing sampling was used to recruit participants. Anonymised interview data was thematically analysed and incorporated Ecological Systems Theory (EST).

**Results:**

Seventeen participants were interviewed (NHS maternity services *n* = 6; local authority *n* = 3 and VCSE *n* = 8). Data highlighted three interlinked levels of barriers that professionals perceived pregnant women living on low income experience accessing maternity care: structural, interactional and individual. Structural barriers included digital exclusion, language-related difficulties and service delivery challenges related to staffing shortages. Interactional barriers included limited social networks, lack of partner involvement, and experiences of racism and discrimination. Lastly, individual level challenges included cost of travel and other pregnancy-related costs, fear of professionals and unfamiliarity with services.

**Conclusions:**

Findings from this study present professionals’ perspectives of the different challenges pregnant women living on low income face when accessing maternity care. These include language and communication, a lack of social support network, the cost and time of travel and the fear of professionals and unfamiliarity of service. Recommendations to improve access to maternity services include the implementation of recycled smart phones, the use of digital translation apps within appointments and the use of pre-paid travel vouchers.

**Supplementary Information:**

The online version contains supplementary material available at 10.1186/s12884-026-08745-7.

## Background

Women living on low income and those from disadvantaged backgrounds experience higher rates of stillbirth, neonatal death, preterm birth, low birth weight, and maternal mortality compared to those from higher socioeconomic groups [1–5]. They also often face multiple, overlapping barriers to accessing maternity care. These include social stressors [6–9], low health literacy [10, 11], limited digital access [12, 13], lack of social and partner support [14–19], language and communication barriers [20, 21], transport difficulties [22–24] and fear of stigma or judgement from healthcare providers [25]. Such barriers result in lower engagement with antenatal care, reduced continuity of care, and poorer experiences of care, with women frequently reporting inadequate communication, feeling disrespected, or not being heard [1, 26, 27]. These challenges are compounded by the intersection of poverty with other social identities such as ethnicity, age, migration status, sexuality, and gender identity [26], which further amplify disparities in outcomes and experiences [2, 3, 28].

While existing evidence documents the experiences of women facing socioeconomic disadvantage, little is known about how healthcare professionals understand and respond to these barriers. Limited research [29–31] has identified the impact poverty, social exclusion and structural inequality has on women’s ability to engage with services from the perspective of healthcare professionals. Research has shown that time pressures, staff shortages, and rigid service models can restrict professionals’ ability to provide continuity of care and build trusting relationships with women from disadvantaged backgrounds [31, 32].

Recent UK policy initiatives, including the NHS Long Term Plan [33], the Women’s Health Strategy for England [28], and the Maternity Disparities Taskforce [34], reflect growing recognition of these inequalities. Together, these policies aim to improve access, quality, and safety for women from minoritised and racialised ethnicities and low-income groups, and to promote equitable, personalised maternity care. However, despite such commitments, implementation has been inconsistent [35], and disparities remain deeply embedded. Alongside this, the digital transformation of UK NHS services, through initiatives such as the Personalised Care Plan [36] and the Fit for the Future: 10 year health plan for England [37], has introduced both opportunities and challenges, with concerns that digital exclusion may further disadvantage women living in poverty.

The Poverty Proofing© approach, developed by the North East England charity Children North East (CNE), provides a model for examining the structural and organisational factors that enable inequalities in maternity care. Starting in schools, initial work in this area focused on ‘Poverty Proofing the School Day’ and was designed to identify and remove exclusionary practices affecting children from low-income families [38]. An evaluation of this approach has shown improvements in attendance, attainment, and inclusion [38]. Adaptions to the approach in healthcare settings have identified a range of barriers experienced by children, young people, and families living on low income [39, 40], highlighting its potential to inform system-level change within maternity care.

This study was informed by the Poverty Proofing© approach, whereby we worked with CNE to consider known structural and organisational factors through their work in schools and other health settings which enabled us to explore local context in more depth. The study aimed to explore professionals’ perceptions of the barriers pregnant women living on low income face in accessing maternity care. Understanding these barriers from the perspective of professionals is essential for developing strategies to enhance the accessibility and quality of maternity care.

## Methods

### Context

Newcastle upon Tyne is ranked 36th most income-deprived of all 316 local authorities in England, with 17.8% of the population income deprived in 2019 [41]. There are 175 neighbourhoods in Newcastle upon Tyne, and 76 were among the 20% most income-deprived in England, while only 34 were in the 20% least income-deprived in England [41]. Newcastle upon Tyne has a large white ethnic population (80%), with ethnic minorities such as Asian and Asian British (11.4%), Black and Black British (3.3%), Mixed or Multiple ethnic groups (2.3%) and any Other ethnic group (3.1%) represented [42]. Newcastle upon Tyne consists of many religions including Muslim (9%), Hindu (1.4%) and Christian (41.3%) [43]. Newcastle upon Tyne NHS Foundation Trust (NuTH) consists of the Royal Victoria Infirmary (RVI), a tertiary teaching hospital with over 8,000 annual births [44].

This was a qualitative study, and participants were recruited for one-off semi-structured interviews (either face-to-face or via telephone/online). Seventeen professionals (NHS, local authority and Voluntary, Community and Social Enterprise (VCSE)) were recruited from NuTH maternity services, local authorities and VCSE organisations located in Newcastle upon Tyne as maternity care often takes place outside of the NHS. Purposeful and snowballing sampling [45] were used following the eligibility criteria as outlined below.

Participants were purposively sampled to provide maximum variation based on the following criteria:

1. Role: Healthcare professionals or professionals working within local authority or VCSE organisations.
2. Involvement: Provides care for pregnant women and postnatal women.
3. Geographical location: working within Newcastle upon Tyne.

Participants were identified from utilising existing contacts of the research team and partners, snowball sampling and poster displays within the maternity unit waiting room in the trust and through VCSE organisations.

Participants who expressed interest in taking part were given a participant information sheet and a consent form. Written consent was obtained before commencing the interview. Interviews lasted between 35 and 60 min using a bespoke topic guide (Supp file 01), informed by existing literature, input from the research team, public members and utilising the six-stages of the Poverty Proofing© approach, with input from Poverty Proofing practitioners, shaping questions to be inclusive of the barriers that stem from poverty and low income. Broad interview topics included: professionals’ roles, their views of the barriers that pregnant women living on low income face when accessing maternity care, and factors that enable good access to maternity services.

Interviews were conducted face-to-face, telephone or via MS Teams by KBT and recorded using a Dictaphone. Recorded interviews were transcribed verbatim by a University approved external transcription company. Data collection stopped when data saturation was reached.

### Theoretical framework and data analysis

Interview data were anonymised by KBT before being imported into NVivo 15 [46]. An inductive reflexive thematic analysis using Braun and Clarke’s [47, 48] six-stage analytical process was undertaken (Stage 1 – familiarisation; Stage 2 – generating initial codes; Stage 3 – searching for themes; Stage 4- reviewing potential themes; Stage 5 – defining and naming themes; Stage 6 – producing the report). Data analysis was undertaken by KBT, KS and DL with input from the research team through regular meetings.

The themes of our findings were informed by Bronfenbrenner’s ecological systems theory (EST) [49, 50]. EST consists of five distinct but inter-related levels of enquiry that explain how individual, organisational and policy level factors influence individuals across their life course [51]. Frequently applied in public health research [51–54], the EST is often adapted to fit distinct research contexts. The EST was adopted inductively during stage 4 of the analysis after review of the initial round of coding revealed multi-level interconnected concepts. This led us to adopt a three-tier level of analysis which spanned: (a) the structural level, which is related to factors that are rooted in broad societal or health policy/immigration policy influences on the experiences of pregnant women; (b) the interactional level, which is focused on factors that are linked with interactions/relationships (of pregnant women) with individuals within the systems with which they engage (such as with family/hospitals/GP clinics etc.), and (c) the individual level, associated with personal characteristics and circumstances of pregnant women (such as the existence of complex needs, fear of professionals, personally experienced challenges related to low income etc.). Using EST allowed for exploration of dynamic interplay between structural, interactional and individual barriers, while explaining how influences at different levels reinforced one another and shaped healthcare professionals’ perceptions of access to maternity care.

### Reflexivity statement

KBT is a physiotherapist and public health researcher trained in qualitative methods, with a keen interest in access to healthcare services. KBT’s professional background and commitment to health equity informed their interest in exploring the barriers to antenatal care. Having previously conducted research with healthcare and VCSE professionals, KBT relied on developing links and connections with local key stakeholders to facilitate introductions. KBT was aware that their position as an academic outside of the study landscape could influence participants’ responses. To mitigate this, KBT ensured analysis was conducted by three members of the research team, with input from the wider research team, and sought validation of preliminary findings via workshops with key stakeholders and public partners. These steps ensured that KBT’s interpretations were grounded in participants’ perspectives.

### Public involvement

Nine public members were involved in the study, providing input into the funding application, assisting in the developing of study materials, recruitment methods and provided input into the language used in study documents e.g. the preference for the term ‘living on low oncome’ rather than poverty to describe our sample. The public members reflected the communities of interest and consisted of women who were pregnant or recently delivered, who lived on low-income and experienced maternity care in the North East of England. Once preliminary themes were developed, four workshops were conducted with members of the research team and six public members (who had recently delivered and were living on low income) and 29 key stakeholders (i.e., healthcare professionals, VCSE representatives and local authority representatives) including three members of the Poverty Proofing team at CNE who inputted into the naming and refining of themes and shaping the policy recommendations seen within the discussion section.

### Ethics approval

Ethical approval for this study was obtained from the Proportionate Review Sub-committee of the London - Surrey Research Ethics Committee (24/PR/0820).

## Results

Interview data were collected from 17 one-to-one interviews with professionals working in an NuTH maternity unit, local authority or VCSE organisations located in the North East of England. Table 1 shows demographic data including age, gender, ethnicity, work setting and length of time working with pregnant women.

**Table 1:** Participant demographic data

Demographic		*N* = 17 (%)
Age (years)	18–24 25–34 35–44 45–54 55+	2 (11.7) 2 (11.7) 8 (47.0) 4 (23.5) 1 (5.8)
Gender	Female Male	16 (94.1) 1 (5.8)
Ethnicity	White British Asian	15 (88.2) 2 (11.6)
Work Setting	NHS Local Authority VCSE organisation	6 (35.2) 3 (17.6) 8 (47.0)
Length of time working with pregnant women (years)	1–2 3–5 6–9 10+	4 (23.5) 3 (17.6) 4 (23.5) 6 (25.2)

Participants described their perspectives on several barriers that pregnant women living on low income experience when attempting to access maternity care. These have been grouped into three overarching themes aligned with EST: (1) Structural factors; (2) Interactional factors, and (3) Individual factors. Illustrative quotes are given below and participants are coded with participant number and profession.

### Theme 1: Structural factors

This theme relates to broader societal and policy factors that influence the experiences of pregnant women, for example health, employment or immigration policies, societal attitudes and prejudices towards certain groups defined by protected characteristics, institutional policies within healthcare providers etc.

#### Digital exclusion and IT

Participants reported that pregnant women living on low income often do not have a smartphone, mobile data, internet access or the financial ability to purchase credit. This led to barriers in accessing BadgerNet notes – an electronic patient records system used by some NHS trusts during perinatal care that comes in the form of an app on smartphones or similar mobile devices. It was reported that when women did not have access to BadgerNet notes this resulted in women not knowing about appointment timings, or how to book, manage or rearrange appointments.

*‘That*,* of course*,* is the other big health inequality that we now struggle with because*,* now that we are supposedly paper-light and everything is on BadgerNet*,* which is absolutely marvellous for the vast majority of people*,* that can be a huge barrier because some people have a smartphone*,* but they haven’t got any data on it. Or*,* if they haven’t got a smartphone*,* they’ve just got a block which*,* obviously*,* they can’t read their BadgerNet notes on. People do lose their phones quite a lot. That happens quite a lot.’ – Midwife (019)*.

#### Language

Language was reported to be a barrier to accessing maternity care particularly for those who were migrants to the UK and for whom English is not a first language. While translation services were used participants reported that often information was lost in translation, hired services at times came across as unprofessional and were difficult to engage with at the correct appointment time.

*‘People with English as a second language*,* I mean*,* it’s a massive barrier*,* isn’t it? The interpretation services that we’ve got aren’t great…. The phone interpreters*,* some of them are very good*,* some of them not so much. You might hear them*,* sort of*,* walking along the street*,* or in the shop*,* doing something*,* and you think*,* “Well*,* actually*,* I want you sitting down*,* listening to what this woman has got to say*,*” as well. So*,* I certainly think that those are barriers.’ – Midwife (020)*.

Participants reported that some women did not want to work with translators as they were fearful of a lack of confidentiality and professionalism. The use of overcomplicated language and medical jargon resulted in many pregnant women asking VCSE practitioners to explain medical letters.

*‘I think*,* yeah*,* the translation they find difficult. I’ve had a few women say that if they had a different set that was in plain English with less acronyms and just better words for things… Like I remember one saying*,* “Uterus?” She was like*,* “If they’d put womb I would’ve known.”’ – VCSE practitioner (011)*.

#### Service delivery – funding and understaffing

Staffing pressures were reported as a barrier for pregnant women to accessing antenatal care. Although healthcare professionals wanted to provide the best possible care, they often felt rushed during necessary clinical procedures, owing to staff shortages.

*‘I think staff shortage when providing healthcare is a big issue. I’ve been speaking to health visiting teams and midwifery teams where there are staff shortages*,* and that obviously puts a strain on themselves*,* where they’re trying to provide the best possible care*,* but it also might mean that it affects mums getting the right support*,* at the right time.’ – VCSE practitioner (010)*.

Participants reported that while support workers were invaluable in providing assistance to pregnant women during appointments, funding cuts impacted their availability. Participants also reported that due to changes in working during the COVID-19 pandemic, working relationships and networks changed, and many had not returned to pre-COVID working conditions, resulting in a lack of awareness of other services and professionals.

*‘I think it became a bit trickier after COVID because people didn’t know each other so well. We’d*,* sort of*,* lost some of those links and contacts a bit. And especially for newer staff*,* they didn’t really have them*,* so it was harder to work out who people could signpost to or whatever.’ – Health visitor (014)*.

### Theme 2: Interactional factors

This theme focuses on the relationships of pregnant women with individuals within the systems that they engage, for example their social networks, families, staff at the hospital and GP clinics, their employer.

#### Social support network

Participants reported that pregnant women who lacked social support from friends and family often found it difficult to attend appointments. Often partners were unable to attend appointments, due to challenges associated with arranging childcare for older children.

*‘At the hospital*,* you can’t bring your children to appointments. So*,* she would either need to find somebody to look after them*,* or the partner needs to stay with them*,* assuming that there is one. So*,* I suppose it’s a barrier*,* because what if she can’t find any childcare? What if she doesn’t want to attend on her own? What if she wants her husband*,* or her mum*,* or someone to come with her*,* but actually*,* she can’t? And with the hospital*,* there’s not always a great deal of flexibility around appointment times.’ – Midwife (020)*.

Participants highlighted that for those pregnant women unable to bring their older children to appointments, and especially those who were single parents, adequate social support was crucial. Some community services explained that they were flexible in allowing a pregnant woman to bring her older children to appointments to ensure that she was able to attend her antenatal care.

*‘Yeah*,* because a lot of them are the*,* like*,* refugees or asylum seekers and don’t really have any family here*,* so don’t really have anyone. It’s just*,* like*,* them and their partners*,* so it is just them*,* so they wouldn’t have anyone else. So*,* for whatever reason if she had to go into hospital or something*,* like*,* she wouldn’t have anyone to watch the kids.’ – Midwife (021)*.

#### Involvement and engagement of fathers/partners

While having fathers and partners involved in maternity care was seen as an enabler, specific barriers preventing their involvement were identified. Working fathers often missed appointments scheduled during work hours, prompting their partners to request appointments outside of working hours.

*‘People will often say*,* “I’d love my partner to come*,* but they’re not going to be able to get time off work*,*” or*,* “It’s more difficult*,*” or*,* “They work away*,*” or*,* “They work shifts.” Or there’s a whole host of reasons that then come into play there*,* but again what we will do there is*,* if they can*,* again we can offer the letter to employer for a partner.’ – VCSE social worker (006)*.

Additionally, participants reported that fathers from different cultures and religious beliefs often felt uncomfortable in maternity and antenatal spaces, as they considered these spaces to be for women only and hence not seen as a space for fathers to attend.

*‘Sometimes dads are there*,* if they can be. Sometimes they prefer not to be. A lot of those cultural things are there as well. So*,* for example*,* a [ethnic group] dad may not want to sit and talk about breastfeeding*,* and all those other things. It may not be appropriate to sit with women and talk about those things. It’s seen as more of the mum’s domain than theirs.’ – Health visitor (014)*.

#### Service delivery - racism and discrimination

Racism and discrimination were reported as a barrier to pregnant women accessing maternity care. Participants reported that pregnant women from minoritised and racialised ethnicities often feel that their needs were unmet due to discrimination based on their race or faith.

*‘So*,* depending on where you’re living*,* and what your surroundings are*,* you might be concerned that your needs won’t be taken into account*,* in terms of the way that you live your life on a day-to-day basis*,* whether that’s to do with your faith*,* or other issues*,* that could be a concern.’ – VCSE practitioner (010)*.

Participants identified that they lacked training in Equality, Diversity and Inclusion (EDI), to ensure that they were able to provide adequate care to those from all ethnicities, and noted that stigma, judgement and staff attitudes were a barrier to women accessing care, due to feelings of being judged for who they are.

*‘So*,* if you’ve experienced judgement or discrimination*,* or you haven’t been helped in the way that you were wanting to*,* that can stop you from accessing further support*,* and during pregnancy*,* it’s a very vulnerable time*,* so that’s another barrier*,* I would say’ - VCSE practitioner (010)*.

### Theme 3: Individual factors

This theme relates to barriers and factors stemming from the personal characteristics and circumstances of the pregnant women, for example their background, existence of complex mental health needs, feelings of anxiety or fear of professionals, personally experienced challenges related to budgeting and low income.

#### Travel related

Several transport specific barriers impacting pregnant women who live on low income from accessing maternity care were identified by participants. The public transport system was reported to be complicated or disjointed and often difficult to navigate by women who may be new to the area.

*‘If they don’t live close by and they don’t have access to a lift or something*,* they’re either not confident enough to use public transport or they don’t know the area well enough to be able to rely on public transport. A lot of them certainly don’t have the money to pay for taxis or anything like that*,* so quite often it is a barrier*,* getting them into our groups – just*,* yeah*,* financially getting to the groups.’ – VCSE practitioner (012)*.

The costs associated with travel were also reported as a key challenge. The fare costs were reported to be high for people living on low income, especially when attending with a partner which doubles the cost. In Newcastle upon Tyne, the lack of a uniform fare across modes of transport (i.e., bus/tram), required multiple tickets increasing overall costs.

*‘And it’s costly. I mean I know it’s capped. But even that- If somebody is going to the hospital*,* even when it’s capped at £2*,* that’s four quid for one person. If you’ve got financial concerns that’s a lot of money. If there are two of you going*,* where do you find that eight quid from? Plus when you get there you might need a drink or whatever. There’s other stuff. Or you might have taken time off work.’ – VCSE practitioner (009)*.

#### Hidden costs

Other associated costs which were not as prominent as the costs of travel but impacted access to antenatal care were also identified by participants included the cost of vitamins and simple medications (i.e., paracetamol or heartburn medication) that GPs would not prescribe, the cost of paid antenatal classes that would take place outside of working hours, as opposed to the free NHS classes that were arranged for during the working day, the cost of attending a larger number of hospital appointments, and finally, the cost of purchasing equipment for the baby (i.e., crib, pram, clothing, formula etc.).

*‘I think it’s just this assumption that*,* “Well-” Because the NHS is*,* obviously*,* free to access. And that’s phenomenal. But I think there is this assumption that*,* “Well*,* be grateful. And we can get you these appointments*,* and that’s great.” But no consideration*,* necessarily*,* of*,* “Well*,* for some people actually being able to access that is almost impossible.”’ – VCSE practitioner (009)*.

#### Fear of professionals and unfamiliarity with service

Participants also reported the perception that there was a fear of professionals and lack of familiarity with maternity services that prevented some pregnant women from accessing care. It was reported that often pregnant women felt anxious about attending a new appointment or group, not fully understanding what it was for, or who would be delivering it.

*‘I think it’s just basic lack of knowledge*,* lack of trust. If they thought they were going to go there and they were going to see their community midwife*,* who they’ve already seen before*,* it might be different but*,* even then*,* you don’t always see the same community midwife now. It’s just another woman in a similar uniform. I can’t imagine what it’s like. These people are doing this stuff*,* you don’t know who they are or what they are.’ – VCSE practitioner (011)*.

Participants reported that women often felt judged on their parenting ability owing to perceived social class and the questions they asked.

*‘Maybe fear of the unknown. “What do I do? Should I ask these questions? If I ask these questions*,* is somebody going to think that I’m not going to be able to take care of the child?” So not knowing what support is available*,* and how that person will be supported.’ – VCSE practitioner (010)*.

For women who had recently migrated to the UK, an additional barrier in the form of unfamiliarity with the process of navigating a healthcare system of which they had limited knowledge and the role of specific professionals within it was also reported.

*‘Sometimes women struggle*,* I think*,* to trust. I think there are issues around trusting. The healthcare’s different from in their country*,* if they’ve had a bad experience before*,* if they’re just not used to accessing different professional services*,* and all of a sudden*,* they have to.’ – Health Visitor (014)*.

#### Prevalence of complex needs

Living with multiple complex needs was reported as a barrier to pregnant women accessing maternity care. For example, professionals reported that pregnant women experiencing domestic violence often missed appointments with phone access controlled by their partners.

*‘Yeah*,* I think there is. I think there are ones that are being controlled and so don’t come*,* and then there are ones who are in those sorts of controlling relationships that they come*,* but the partner takes over the appointment. That can be quite tricky’. – Midwife (019)*.

Similarly, those who had experiences of drug and alcohol use were reported to need greater input into their care to facilitate their attendance in antenatal appointments. Professionals noted that for those with complex needs, additional training in trauma-informed care would be beneficial.

*‘I don’t know*,* the chaos in some households and then you’ve got things… you’ve got addiction*,* you’ve got ADHD*,* domestic abuse*,* homelessness.’ – VCSE team manager (013)*.

Housing was often cited as a key cost as there was a rise in pregnant women requiring housing assistance owing to poor housing conditions, homelessness or overcrowding. It was reported that women often disengaged with services due to the emotional and cognitive toll of having poor and inadequate living conditions.

*‘Housing is a big one. We’ve definitely seen an increase in housing issues in the past year. I think the current situation with housing in [CITY] is definitely having an impact.’ – Midwife (018)*.

Additional quotes that informed policy recommendations can be found in Supp File 02.

## Discussion

The findings from this study present professionals’ perspectives of the different challenges pregnant women living on low income face when accessing maternity care. Three levels of barriers, informed by EST, across the structural, interactional and individual levels, were identified through thematic analysis of the interview data, collectively hinder access to maternity care.

Structural barriers included digital exclusion and language related challenges. With a growing demand to go ‘paper-free’ there has been an increase in the use of digital technologies within healthcare [36, 37], such as BadgerNet notes. Our study shows that professionals are aware that the use of these digital technologies can be exclusionary and difficult to navigate for those who do not have access to smart phones, data or WiFi. This confirms previous systematic review findings [12, 13] which confirmed large disparities in digital access and digital literacy that resulted in a reduction in accessibility of services, and called for the reduction of digital inequalities. Language was another structural barrier identified, with participants reporting challenges in communicating with women for whom English was not their first language, with concerns over the quality and accessibility of interpretation services. These findings corroborate previous research [21], that found that it was often difficult to access interpreters when needed and that the quality of the interpretation was sometimes questionable.

Two factors that drove interactional level barriers were related to limited social networks and non-involvement of fathers/partners. Corroborating previous research [14–16], participants reported that pregnant women who had a limited social support network of friends and family often engaged less with maternity care, frequently citing a lack of childcare and feelings of isolation, especially among those new to the country. Limited involvement of fathers owing to their inability to get time off work to attend appointments during work hours, or cultural barriers which consider maternity care to be exclusively for women, were also identified as a barrier. These barriers also align with previous studies that have highlighted similar issues in effectively engaging with fathers [17–19]. It is important to note that racism and discrimination was acknowledged as a barrier to accessing care, in our majority white sample. It was identified that their own lack of EDI training, stigma and judgement of ethnic minority women acted as a barrier to providing safe and supportive care. An area that they identified could be strengthened.

Lastly, individual level barriers were related to travel costs and unfamiliarity with maternity services. Travel-related challenges were a major barrier that was cited by most of the study participants. Consistent with previous research [22, 23], the public transport system was reported as costly, complicated, unreliable, and difficult to navigate, especially for women new to the area. Another barrier specific to individual circumstances is the unfamiliarity with services and fear of professionals which often caused anxiety among pregnant women when attending new appointments. They also feared being judged due to their socioeconomic circumstances or for asking questions, worrying that professionals would perceive them as unable to care for their baby. This unfamiliarity with the healthcare system was exacerbated for women from a migrant background, who lacked a point of reference for navigating the NHS due to the differences with their home country’s healthcare system [1, 24].

These barriers did not operate in isolation but intersected across the levels. Professionals reported that for many women living on low income, individual barriers like travel difficulties and an unfamiliarity of services were intensified by interactional challenges like limited social support or discriminatory practice, while also being shaped by structural barriers including digital exclusion and language. These interacting barriers create disadvantage for pregnant women living on low income, resulting in poor access to care and professionals’ capacity to provide equitable care.

### Policy Recommendations

Our study findings could inform policy recommendations on reducing the barriers to accessing maternity care for women living on low income and build on recommendations made in a recent umbrella review of interventions [55]. We recommend providing recycled smart phones and pre-paid Sim cards to those who require digital access, the use of translators in all appointments while reviewing the professionalism of these services and starting to include translation apps to support appointments. We recommend implementing free travel on public transport for women and their partners on days of appointments, using pre-paid travel vouchers, and providing appointments outside of normal clinic hours to support engagement from partners who are unable to get time off work. Finally, we recommend that professionals delivering care to pregnant women are provided EDI, cultural sensitiveness and trauma informed training to further support women and families. While workplaces develop detailed referral pathways to local VCSE organisations and policies to enable effective use of translators and additional care pathways for those with complex needs. These policy recommendations stem from the interview data and refined within the four workshops that were delivered with public members and key stakeholders. The Poverty Proofing© approach informed data interpretation, with key stakeholders from CNE present at the workshops to help shape with recommendation development.

### Strengths and Limitations

This is the first qualitative study exploring professionals’ experiences of the barriers to accessing maternity care for women living on low income. Participants were professionals working in the healthcare, local authority or VCSE sectors and provide valuable perspectives into the experiences and challenges pregnant women living on low income experience when accessing maternity care. As this study reflects the perspectives of majority White professionals rather than women living on low-income, this will shape how the barriers are understood and interpreted. Our data analysis process was rigorous and involved our data being analysed by three researchers, with input from the rest of the research team through regular data analysis meetings. We built upon this and conducted four stakeholder and PPI workshops to confirm themes derived. A wide range of VCSE organisations were identified, through which professionals were recruited, allowing for different perspectives to be shared. Finally, using EST enhanced data interpretation by allowing examination of the interactions between individual, service level and structural barriers, thereby facilitating a deeper understanding of how barriers experienced by women shape access to maternity care.

Recruitment was focused within the North East of England, which is a limitation. The area covered by these organisations is urban, and therefore the perspectives of those who work in rural or coastal areas were not captured in this study. Other geographical areas may have additional policies and services for supporting women living on low income not present in our study’s location which may benefit those living on low income.

## Conclusion

This study provides vital insight and professional perspectives into the barriers and challenges that pregnant woman living on low-income experience when accessing maternity care. Our interview data identified several structural, interactional and individual barriers to accessing care, including difficulties with digital technologies, costs of travel, language, a fear and unfamiliarity of services and professionals and a lack of social support, showing that professionals are aware of these barriers and the impact they have on woman engaging with services. Our findings can inform actionable service and policy recommendations to assist in overcoming of these barriers to care. Finally, considering the Fit for the Future: 10 years health plan for England, additional research needs to explore the impact digital technologies in maternity healthcare play on women accessing maternity care, and evaluating any interventions that aim to reduce barriers to accessing care for women living on low income.

## Supplementary Information

Supplementary Material 1.

Supplementary Material 2.

## Data Availability

The datasets generated and analysed during the current study are not publicly available due to the sensitivity of the topics discussed within the data but are available from the corresponding author on reasonable request.
