# Supplementary material for: Professional perspectives on barriers to accessing maternity care in England: a qualitative study
Source: BMC Pregnancy Childbirth. 2026 Feb 10;26:334. doi: 10.1186/s12884-026-08745-7 (PMC13020039; doi:10.1186/s12884-026-08745-7)
Supplement: Supplementary file 1 — Supplementary Material 1. [file 12884_2026_8745_MOESM1_ESM.docx]

Supporting Engagement with and Access to Maternity Services: a qualitative and survey study

WPA Healthcare, voluntary and community group professionals interview topic guide

Researcher introduces themselves, and reminds participant of confidentiality.

Thank you for agreeing to take part in this interview, and for giving up your time. We are inviting a number of healthcare/community/voluntary group professionals from the North East of England to take part, so I am pleased that we are meeting today.

The premise behind this research is that pregnant people living on low incomes (The most commonly used definition of poverty or low income is when someone lives in a household with an income below 60% of the current median household income, taking into account the number of people living in the household (Joseph Rowntree Report, 2022) are at an increased risk of having an adverse pregnancy outcome and challenging social circumstances may further impact their mental health and wellbeing.

However, despite the increased risk of adverse pregnancy outcome and mental health issues, pregnant people living on low incomes are less likely to access and engage with maternity services. This may be in part due to the costs associated with being pregnant and accessing maternity services.

This research aims to identify and explore the barriers to accessing maternity services experienced by pregnant people living on low incomes, and identify a range of practical solutions/actions that maternity services can adopt that will increase access to these services.  So, we are specifically interested in pregnant people who are living on low incomes rather than all pregnant people.

We are interested in finding out about your experiences and perspectives of supporting pregnant people on low incomes during pregnancy, to better understand what could be done to address some of the inequalities and overcome some of the barriers to accessing care.

The interview will last no longer than an hour and there will be an opportunity for you to provide any further information that has not been covered. Let me know if you would like to have a break or stop the interview at any point. Feel free to ask questions at any stage during the interview.

**YOUR ROLE AND INVOLVEMENT:**

**These first few questions are going to be around your role and involvement in supporting pregnant people during pregnancy:**

*Role*: Could you tell me about your current occupation and responsibilities?

*Setting:* Could you provide some information about the setting that you work within (i.e., healthcare/community/maternity)?

*Involvement*: In what ways are you involved in supporting pregnant people or people who had recently gave birth?

Do you talk to pregnant people about any aspects of their pregnancy experience (or postnatal) other than their health?

What other aspects of a pregnant persons life do you talk about? (i.e., housing, finance, worries)

How do you approach these topics with pregnant/postnatal people?

Do you ever ask a pregnant people if she has any financial worries?

How confident do you feel in asking this type of question, and having those discussions?

If yes, where do you signpost these people to?

If no, why not?

What services are available to pregnant people?

How do you inform pregnant people of the many different services available to them throughout their pregnancy?

Where does any financial advice and support currently come from along the maternity pathway?

**BARRIERS:**

**These next few questions are going to be around the barriers faced by pregnant people living on low incomes to accessing maternity care:**

What challenges are you aware of for pregnant people to access maternity services and care?

From your point of view, what do you think are the main challenges faced by pregnant people living on low incomes?

What do you see as some of the barriers faced by pregnant people and their partners/families who are living on low incomes when they are trying to access maternity services?

How do you try to overcome these difficulties and challenges within your work and role?

What more do you think could be done to overcome some of these challenges for pregnant people?

What challenges do you face in supporting pregnant people?

What resources do you currently have access to or are aware of that support pregnant people living on low incomes?

What resources would help you to be able to better support pregnant people living on low incomes?

What resources do you feel need to be made available that may not have been?

What do you think can be done to improve access to maternity services in particular for those living on low income?

What additional costs do you think are associated with pregnancy and accessing maternity care? (i.e., social; time off work; travel; parking etc)

Could you talk me through some of the other barriers that pregnant people face when accessing maternity services at your hospital?

Do you feel there is anything that you and your colleagues can do to ensure better uptake of maternity services? Anything that you would change?

**ENABLERS:**

**These next few questions are going to be around the enablers available to pregnant people living on low incomes to help accessing maternity care:**

Is there any national and regional/local services/resources that you know of that help pregnant people living on low income? Are you able to talk me through what these are, and what they look like?

Are you aware of any social networks in the area that are helpful to pregnant people living on low income? How do you signpost to these services?

Are you aware of any resources that are currently available to pregnant people to help them financially during their pregnancy? Can you talk me through what these services look like and what they entail for the pregnant person?

Have you seen or heard of any services that have changed the way they deliver their services to help those who are living on low income to access services?

What needs to be done to ensure that pregnant people living on low incomes engage with maternity services?

**ANY FURTHER INFORMATION:**

Anything else that you would like to add that has not already been discussed?

Do you have any questions?

Remind the participant about confidentiality

Thank you for your time and involvement.
