# Supplementary material for: Professional perspectives on barriers to accessing maternity care in England: a qualitative study
Source: BMC Pregnancy Childbirth. 2026 Feb 10;26:334. doi: 10.1186/s12884-026-08745-7 (PMC13020039; doi:10.1186/s12884-026-08745-7)
Supplement: Supplementary file 2 — Supplementary Material 2. [file 12884_2026_8745_MOESM2_ESM.docx]

Supp file 02 – policy recommendations and quotes

| **Policy recommendation** | **Quote(s)** |
| --- | --- |
| 1. Digital access | ‘“Oh, well, buy people iPhones,” but I don't think that's safe in the population that we're looking after. In social care, we’re providing burner phones, but it is a case of, like, missed and broken phones.’ – Midwife (018)  ‘We can now provide a SIM card for a year. So, obviously, they’d need the handset, but we can provide a SIM card for a year. And I think she said, has it got 1mb or 2mb, or gig, or something, of data.’ – Midwife (020) |
| 1. Translators and interpreters | ‘So, it’s really giving that information to parents, in their language, in their home language, whether it’s literature that’s written, or spoken, or via a translator, and interpreter.’ – VCSE Practitioner (005)  ‘And I think health professionals have a duty to present information that’s in an inclusive way, to do that. However, the barriers are that the appointments are really short and midwives don’t have the time…. But, as I said, the onus then is on the health professional to make this more accessible and understandable.’ – VCSE Practitioner (009)  ‘Because although sometimes people have family or friends who can interpret, it’s not always suitable or appropriate, and I think it’s important that we have funding for that.’ – VCSE Practitioner (010)  ‘So I try and, like, bring up little posters and leaflets and things, so they can have something visually to look at. And, like, try and have that translated through an interpreter’ – Midwife (021) |
| 1. Public transport | ‘actually maybe a ticket to get to the appointment, it’s only used- it’s like a library ticket, only used for those, and maybe it gets stamped. That parent has attended that appointment. So, it’s giving you also the incentive to get to your appointment, but also, that you’ve got there and it’s been legitimately used, and you haven’t just gone to the movies and come back.’ – VCSE Practitioner (005)  ‘Or I would just like it if pregnant women can have a free bus pass. You know? Something that makes navigating it easier. A free travel pass.’ – VCSE Practitioner (009)  ‘I know that when I used to work at Citizens Advice, if you were on income-based benefits, you could get access to free transport around the area, which is fantastic, and that’s really helpful’ – VCSE Practitioner (010)  ‘if the woman can pay to get the bus, if she provides a ticket, we can give her the money back.’ – Midwife (020)  ‘So, like I say, free travel to appointments would make things a lot easier for families’ – VCSE Practitioner (004) |
| 1. Appointments outside of clinic hours | ‘just having that flexible working, like, the extended hours would definitely help. Just to, like, fit more women in and get the woman to come, like, at a time that suits them. Because obviously we’re trying to, like, give them appointments that are more for us and not really beneficial for the woman and the families.’ – Midwife (021)  ‘even though they have appointments on different days, the actual flexibility of appointments for the hospital doesn’t necessarily work’ – Midwife (020) |
| 1. EDI and cultural sensitiveness training | ‘Give us more training on what is available for what sort of aspect of woman. So, “Right, if you’re British and you’re born here, this is what you’re entitled to. If you’re a student from Nigeria, this is what you’re entitled to,” so that when we see these women, I think, “Actually, I do know what I can help you with.”  Because, I had one woman asking if I could get her a bus pass, and this was before I’d spoken to [FEMALE COLLEAGUE], it was probably a year ago, and I thought, “I haven’t got a clue, absolutely no idea,” you know? (Laughter) Now, as it happened, that was the time that interpreter knew how to do that. And again, a lot of the stuff needs to be done through the Universal Credit portal or the Jobcentre.’ – Midwife (020)  ‘You have to go through official training, and safeguarding’ – VCSE Practitioner (013) |
